# Two-Dof Upper Limb Rehabilitation Robot Driven by Straight Fibers Pneumatic Muscles

**DOI:** 10.3390/bioengineering9080377

**Published:** 2022-08-09

**Authors:** Francesco Durante, Terenziano Raparelli, Pierluigi Beomonte Zobel

**Affiliations:** 1 Department of Industrial and Information Engineering and Economy (DIIIE), University of L’Aquila, P.le Pontieri 1, Località Monteluco, 67100 L’Aquila, Italy; 2 Department of Mechanical and Aerospace Engineering (DIMEAS), Politecnico di Torino, Corso Duca degli Abruzzi 24, 10129 Torino, Italy

**Keywords:** rehabilitation robot, upper limb, straight fibres pneumatic muscle, fuzzy control

## Abstract

In this paper, the design of a 2-dof (degrees of freedom) rehabilitation robot for upper limbs driven by pneumatic muscle actuators is presented. This paper includes the different aspects of the mechanical design and the control system and the results of the first experimental tests. The robot prototype is constructed and at this preliminary step a position and trajectory control by fuzzy logic is implemented. The pneumatic muscle actuators used in this arm are designed and constructed by the authors’ research group.

## 1. Introduction

The continuous growth of average lifespan in the world means more elderly people in the future. That is why more and more sanitary care with a growing of the health cost is expected. This is the main reason that pushes the development of automated systems to apply medical therapies. The physical rehabilitation sector is a very expensive sector because the main part of the therapy has to be performed with one-to-one attention from a therapist. The rehabilitation robots permits for economizing medical therapists, which apply the therapy on a person-to-person basis.

On the other hand, robots are increasingly present in daily life, from robots for cleaning the house, to robots for garden care or self-driving vehicles, etc. As the number of applications useful in normal daily life grows, the need for integration in domestic environments and the need for safety in the interaction between man and machine grows as well. In this context, in recent years, collaborative robots and soft robotics have received a lot of attention, which meet these needs not only in biomedical and industrial fields, but also in the field of exploration and cooperative human assistance [1,2,3,4,5,6,7,8].

In the category of machines with high safety requirements, robots for motor rehabilitation and aid are certainly included.

There are two broad categories of active rehabilitation machines based on the way of mechanical interfacing with humans. There are end-effector type machines, which work by being in contact only with the extremity of the limb to be treated [9,10,11,12,13,14,15,16,17]; and exoskeleton-type machines or devices with a mechanical structure that mirror the skeletal structure of the limb, i.e., each segment of the limb associated with a joint movement is attached to the corresponding segment of the device [18,19,20,21,22,23,24,25,26,27].

Bioinspired machines are more easily placed in a domestic context and are more easily accepted from a psychological point of view. The exoskeleton-type machines are certainly bioinspired whereas the end-effector ones often derive from the adaptation of industrial robots. To ensure safety, these robots must be equipped with systems to introduce compliance. This can also be obtained through control but it is not always possible, for example, when the present transmissions do not allow backdriveability.

The exoskeleton-type machines also allow you to control the individual joints and guide the limb with precision in complex movements. The end-effector type machine is easier to use but may have critical issues related to the achievement of singularity configurations of the human limb. Among the kinematic architectures for end effector type systems, there are widespread projects with cables that allow quite easy control but are bulky and difficult to transport as machines. In [28] an active rehabilitation robot, for the upper limb, a parallel kinematic structure is proposed.

The present work in particular deals with a robot for upper limb rehabilitation. Robots for motor rehabilitation of the upper limb have been studied and used for some time. As for the actuators, they can play a fundamental role in safety. In general, but in particular for robots for rehabilitation or upper limb aid, by far the most used are electric actuators [29,30,31,32,33,34,35,36,37,38,39,40] but pneumatic actuators [41,42,43,44,45,46] and hydraulics [47,48,49,50] are also used or based on magnetorheological [51,52], electro-rheological [53] materials or with passive elastic elements combined with electric motors and functional electrical stimulation (FES) [54] or FES alone [55].

In the present context, pneumatic muscle actuators, although used very little for rehabilitation devices in general, and in particular for devices dedicated to the upper limbs, are very interesting, given their peculiar characteristics particularly suitable for these devices [56,57,58,59,60,61,62,63]. In fact, they have a great power-to-weight ratio, and they are light, which allows for developing easily transportable or wearable devices. In addition, they are cheap, flexible, and therefore easy to place in the context of the machine, not requiring precision in assembly. Above all, they are compliant; this feature makes the whole machine compliant and therefore intrinsically safe. In the face of these advantages, they have the disadvantage of being more difficult to control than other actuators since they have highly non-linear behavior. This is also the reason why sizing is more difficult. Today, several procedures and models are available for sizing pneumatic muscles [64,65,66,67,68]. Furthermore, they only work in one direction and therefore must be organized in agonist-antagonist architectures. However, this brings another advantage, namely the similarity with man in appearance and operation, which makes it more acceptable and better suited in a domestic context. Furthermore, the compliance, together with the antagonist agonist configuration, allows for having variable stiffness of the joints [69].

The robots for upper limb rehabilitation have different characteristics, especially for the possibilities of exercises they allow. There is no standard on performance and the various devices are distinguished not only by the architecture (exoskeleton or end effector), but also by the joints or movements they can handle. There are robots for the rehabilitation of the shoulder [70], the elbow [71,72,73], the forearm [74], the wrist [75,76] or of the fingers [77,78,79], or for the rehabilitation of numerous joint combinations, such as shoulder and elbow [80,81], forearm and wrist [82,83], wrist and fingers [84], shoulder elbow and forearm [85], elbow forearm and fingers [86], forearm wrist and fingers [87], or whole limb [88].

Control is a key part of rehabilitation robots. First of all, the case in which the control must introduce compliance must be considered. Classic control strategies such as PID control are often used which can work well in the case of passive patient protocols. Other control systems used are those based on sliding mode, mechanical impedance control or fuzzy logic [89], or in combination with each other. Control systems usually use EMG signals [90,91], signals from measurements of kinematic parameters [92] or of dynamic parameters, or in combination [93].

On the basis of all the literature analyzed, an activity was carried out, which is presented in this work, concerning the development of a robot for the rehabilitation of the upper limb for the treatment of the shoulder and elbow with a kinematic architecture that can be seen both as an end-effector and as an exoskeleton type. In fact, the robot, although it is expected to have its own end-effector as its only connection point with the user’s hand, has an anthropomorphic architecture with joints and segments homologous to those of the human limb. It is a device with two motorized degrees of freedom (D.O.F.), actuated by pneumatic muscles, and is particularly innovative from this point of view because the muscles used are of the Straight Fibres type that have several advantages over the McKibben muscles. After the design phase, the robot was built. A control system based on Fuzzy logic has been implemented and, at the moment, the operation isokinetic mode, with passive patient, has been implemented. Some preliminary experimental tests, concerning step movements of the single joints, trajectory tracking of the single joints and trajectory tracking involving both joints at the same time, have been carried out and documented. Tests prove the validity of the project.

## 2. Materials and Methods

### 2.1. Mechanical Design of the Robot

#### 2.1.1. Technical Specification, Functional Design

As previously explained, the robot is designed to use for rehabilitation of upper limbs. A survey that involved users and therapists in order to determine the desirable specifications for an upper limb motor rehabilitation machine resulted in a machine for therapies in the home environment and with characteristics that fall into four categories [94]: individualized theraphy, or the possibility to personalize the therapy for the user, movement and task, i.e., the movements and tasks that can be carried out, recording of performance or everything related to the possibility of documenting progress over time, and safety and usability, i.e., all the relevant characteristics concerning safety.

In particular, the machine must be transportable and therefore light, with a small footprint on the ground (safety and usability). Therapies should primarily focus on the movements of normal Activities of Daily Living (ADL). From an analysis of the ADLs, the main movements involved are flexion-extension of the elbow, prono-supination of the forearm, and flexion-extension of the shoulder (movement and task). Other key features for a rehabilitation machine are safety (safety and usability) and user acceptability (safety and usability). It must be adaptable to a large number of users (individualized therapy), able to record and monitor the user’s performance (recording of performance), and have a user friendly interface (safety and usability). Finally, it must be low-cost. The acceptable cost should be EUR 5000.

Therefore, the technical specifications that were considered for the robot design are:

- rehabilitation with movements in the sagittal plane: flexion and extension of the elbow and flexion and extension of the shoulder in a physiologically correct way or movements that involve all joints at the same time;
- 2 modes of functioning: passive and active-constrained;
- good compliance for safety purposes;
- weight, not more than 400 N;
- cost, around EUR 5000;
- footprint, 600 × 800 mm^2^;
- friendly interface;
- good acceptability by the user.

**Figure 1:**
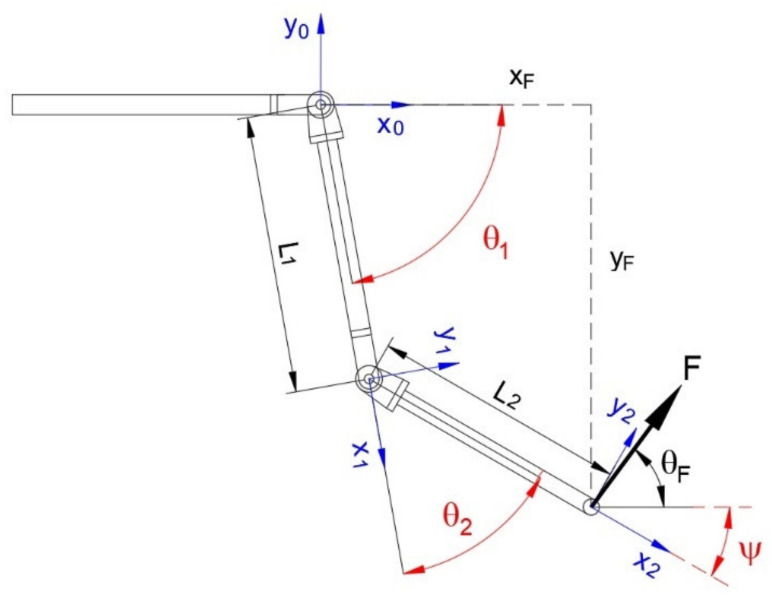
Geometrical parameters of the two links and local references and parameters for kinematic analyses.

Regarding technical specifications, the conceptual phase proposed an anthropomorphic system that operates in a position parallel to the user’s arm, with 2 dofs, one for the shoulder and one for the elbow. Moreover, the machine has to be able to apply a force F = 20 N in any direction to the user’s arm. The anthropomorphic structure gives a better functionality at the robot and the 2-dof promises a better performance in the physical rehabilitation if compared with 1 dof [89]. The dimensions are comparable with those of the human arm according to the following parameters (Figure 1):

- arm length L_1_: 435 mm;
- forearm length L_2_: 385 mm;
- shoulder excursion −110° < θ_1_ < 90°;
- elbow excursion 0° < θ_2_ < 160°;
- direction of force on the end-effector 0° < θ_F_ < 360°.

As for control, this must guarantee stable and extremely robust dynamic functioning of the machine with respect to the uncertainties of contacts in interactions with humans, therapists, or users. It must modulate the response to mechanical perturbations and ensure a gentle and soft evolution both for safety reasons and good therapeutic practice.

#### 2.1.2. Direct Kinematic Model—Kinematic Domain

For the determination of the working volume, the direct kinematic model is considered. Using the Denavit–Hartemberg notation, the transformation matrix between the reference frame of the end link with respect to the base is given by the product of all the single transformation matrices between link i and link i-1:

(1) T20=T10 ⋅ T21=cosθ1−sinθ10L1cosθ1sinθ1cosθ10L1sinθ100100001⋅cosθ2−sinθ20L2cosθ2sinθ2cosθ20L2sinθ200100001=cosθ1cosθ2− sinθ1sinθ2−cosθ1sinθ2 − sinθ1cosθ20L2cosθ1cosθ2− L2sinθ1sinθ2+L1cosθ1cosθ1sinθ2+sinθ1cosθ2cosθ1cosθ2− sinθ1sinθ20L2cosθ1sinθ2+L2sinθ1cosθ2+L1sinθ100100001

**Figure 2:**
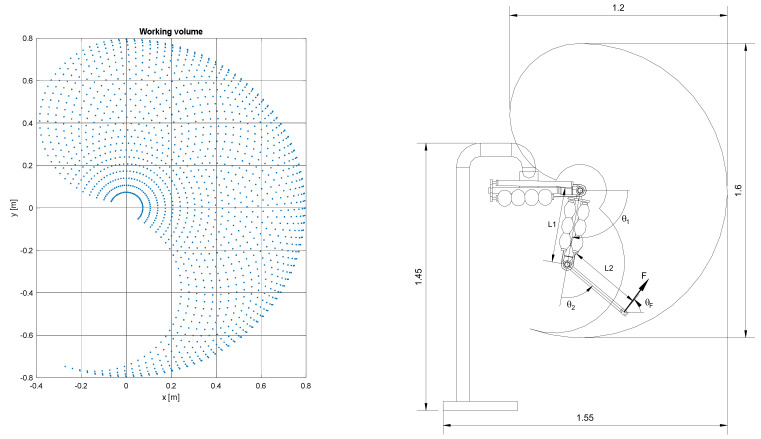
Kinematic domain of the robot and overall dimensions.

By this matrix, it is possible to determine the coordinates, with respect to the base, of any point, known as its coordinates with respect to the local reference of the end link, for any pair of joint angle values. Using the position of the end of link 2 in local coordinates (**p** = [0 0 0 1]^T^) and by varying the angles of the joints in the respective definition domains, the working volume of the robot is determined. In Figure 2, the working volume is presented, with variations of 5° for θ_1_ and θ_2_, obtaining 1435 different positions.

#### 2.1.3. Mechanical Load Model

In order to determine the required torque at the joints for the different operations, a dynamic model has to be considered.

Since the machine must operate at low speeds (max. 100 mm/s), a kineto-static model has been considered. This was obtained by the Eulerian approach based on free body diagrams. Below is the considered model:

(2) $$T_{1\;}{=m}_{1}\cdot g\cdot \frac{L_{1}}{2}\cdot {\cos\theta }_{1}{+m}_{j2}\cdot g\cdot L_{1}\cdot {\cos\theta }_{1}{+m}_{2}\cdot g\cdot \left(L_{1}\cdot {\cos\theta }_{1}+\frac{L_{2}}{2}{\cos\theta }_{2}\right)+F\cdot {\sin\theta }_{F}\cdot {(L}_{1}\cdot {\cos\theta }_{1}+L_{2}\cdot \;{\cos\theta }_{2})-\;F\cdot {\cos\theta }_{F}\cdot {(L}_{1}\cdot {\mathrm{sen}\theta }_{1}{+L}_{2}\cdot {\sin\theta }_{2}{)+m}_{m}\cdot g\cdot {(L}_{1}\cdot {\cos\theta }_{1}{+L}_{2}\cdot {\cos\theta }_{2})$$

(3) $$T_{2}{=m}_{2}\cdot g\cdot \frac{L_{2}}{2}{\cos\theta }_{2}-\;F\cdot {\mathrm{sen}\theta }_{F}\cdot L_{2}\cdot {\cos\theta }_{2}-\;F\cdot {\cos\theta }_{F}\cdot L_{2}\cdot {\mathrm{sen}\theta }_{2}{+m}_{m}\cdot g\cdot L_{2}\cdot {\cos\theta }_{2}$$

where

- T_1_ = torque required on joint1
- T_2_ = torque required on joint2
- m_1_ = mass of link1 (arm) = 2 kg
- m_2_ = mass of link2 (forearm) = 0.45 kg
- mj_2_ = mass of joint2 = 2 kg
- m_h_ = mass of handle = 0.1 kg.

A complete multivariate investigation was performed, by this model, on the parameters θ_1_ θ_2_ ed F according to the values indicated above and it was possible to determine the trends of the torque required at the joints as a functions of the joint positions. Table 1 shows the maximum and minimum values of the required torques.

#### 2.1.4. Actuators, Transmissions—Technological Specifications

About the actuation of the joints, pneumatic muscles were chosen in an agonist–antagonist arrangement. A pulley teeth belt transmission is used for this purpose. As for the risk of transmission slippage, this is covered by the use of RPP type belts with a parabolic profile of the teeth suitable for the transmission of high forces and by an ever-present tensioning by the agonist–antagonist action of the actuators. As for strength, the belts chosen have fiberglass reinforcements with a protective nylon filter. With regard to the requirements of the actuators, force and linear range, once the necessary torques for the joints have been determined by the kineto-static model, the diameter of the transmission pulleys must be chosen in order to determine the specifications of the pneumatic muscles. The pulley must be chosen considering two conflicting needs. As the diameter increases, the forces required by the muscles decrease but their strokes increase. Therefore, the dimensioning of the muscles together with the transmission is a single process.

As already introduced, it was decided to use the straight fibers pneumatic muscle actuators designed and manufactured by the authors [95,96]. The straight fibers pneumatic muscle consists of a rubber tube with a certain number of threads placed inside the wall in the axial direction. The tube is attached at either end to fittings. Some annular rings are positioned along the tube to stop the deformation in the corresponding section. These rings subdivide the tube into 3 or 4 segments. Three materials are used in the muscle: a silicon rubber for the tube, glass fiber for the threads, and aluminium for the fittings. The behaviour of this actuator is strongly non-linear because of the non-linear σ-ε rubber relationship and because of the operative large deformations. The pneumatic muscle actuators provide high safety because of their compliance. This type of actuator has a better behavior than the McKibben muscle as it has no sliding parts in contact, which are a source of energy dissipation and wear. Furthermore, the muscle with straight fibres, during operation, occupies a certain radial volume which can act as a protection system from the rigid parts of the machine.

The dimensioning of the actuators has been addressed by means of the procedure proposed by the authors and described in [95].

**Figure 3:**
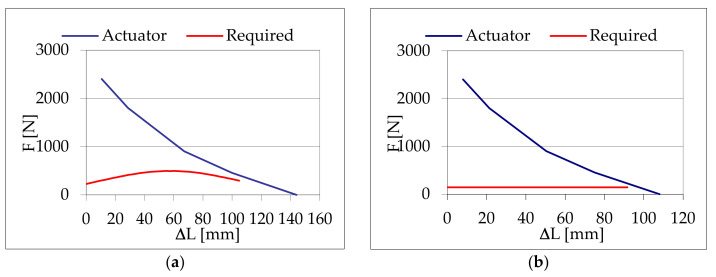
Muscles characteristics obtained by design procedure compared to those required by the machine: (**a**) shoulder joint, (**b**) elbow joint.

Two couples of muscles drive each of the two joints of the robot, shoulder, and el-bow with a diameter of the transmission pulley for both joints of 63.66 mm. Table 2 reports the functional characteristics of a single muscle used in the 2 joints, and Figure 3 shows the relations traction force vs. contraction for the single muscle used compared to the respective required characteristic for the joints. The working maximum operative pressure of the pneumatic muscles used is 0.24 MPa.

#### 2.1.5. Detailed Design

**Figure 4:**
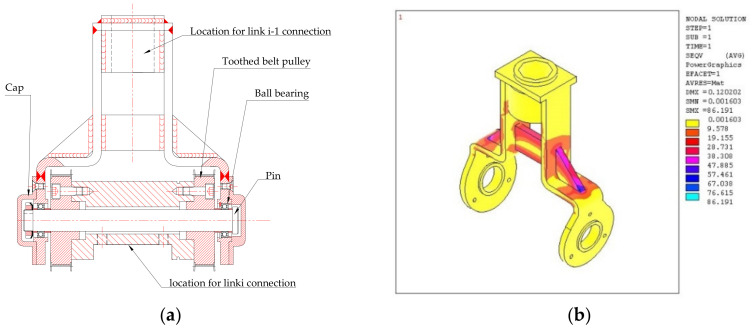
(**a**) Draft of the fork. (**b**) Example of output of the finite element analysis of the fork.

Other aspects of the design are explained in the following. The robot is installed firmly on a steel vertical rod, and the height is fixed considering that the patient will be seated in an armchair for the therapy. The articulation between the robot links is carried out by means of a fork (Figure 4a) made by bent and welded steel. 

The calculation of the fork was made using a numerical modelling by the Ansys finite element code, Figure 4b. Two forks are used in the robot, one for each joint. Each fork is coupled with a cylinder by two ball bearings to make up a hinge. Two pulleys are fixed at the ends of the cylinder.

The structure of arm is made by a tubular element of aluminium. At one end, the tube is linked to the fork as part of the elbow joint, whereas at the other end, it is coupled with the cylinder as part of the shoulder joint. The 4 muscles that drive the elbow joint are placed on the arm. Each of these muscles is linked at one end with the belt, whereas the other end is linked at a plate fixed on the structure of the arm. Additionally, the structure of the forearm is an aluminium tube. At one end, the tube is linked at the fork as part of the elbow joint, whereas at the other end, it has a handle made by a simple aluminium tube. The 4 muscles that drive the shoulder joint are placed on the fixed structure. Angular position transducers (potentiometers) are installed coaxially to the hinges of the joints.

**Figure 5:**
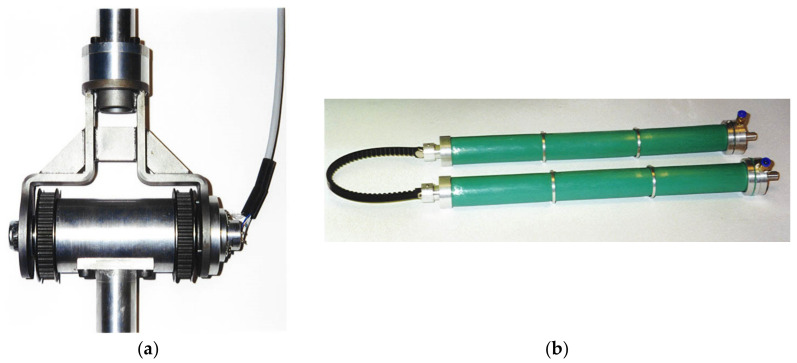
The execution of the joint (**a**). Two pneumatic muscles connected to the tooth belt in agonist–antagonist arrangement (**b**).

**Figure 6:**
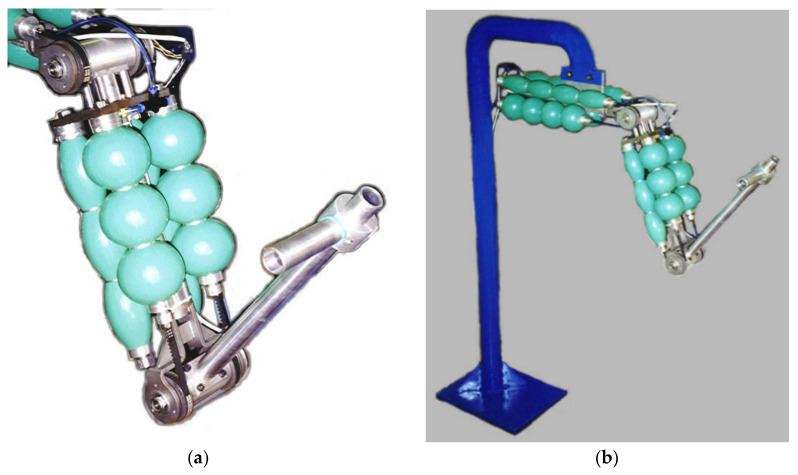
Particular of the arm with the elbow in a flexion configuration (**a**) and an overall view of the prototype of the rehabilitation robot (**b**).

A picture of the execution of one joint is shown in Figure 5a. In Figure 5b, a view of two pneumatic muscles connected to the tooth belt ready to be mounted on the robot are shown. Figure 6a shows the particular of the elbow joint of the robot arm in a flexion configuration and, in Figure 6b, the overall view of the robot is presented. 

### 2.2. Control System

#### 2.2.1. Hardware

**Figure 7:**
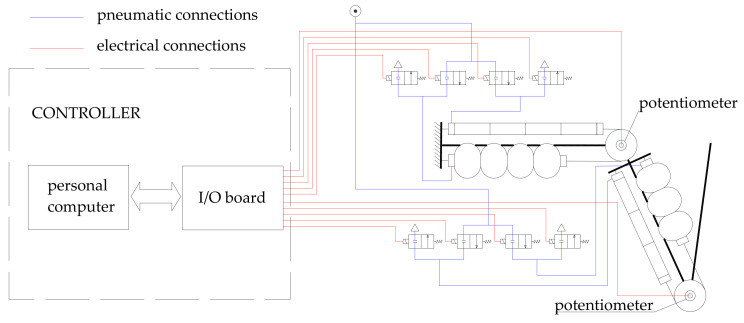
Control hardware.

As said before, every joint is driven by two couples of muscles working in parallel: the agonist couple and the antagonist couple. Supply and exhaust of the muscles are provided in two ways by two positions of high frequency Pulse Width Modulation (PWM) driven digital valves manufactured by Matrix SpA. The digital valves are driven by a data acquisition board by National Instruments on a PC according to the scheme in Figure 7. 

The control system can adjust the air mass entering the muscles on the basis of the feedback signals given by the rotation of each of the two joints, measured by a conductive plastic potentiometer. This is a precision potentiometer with an electric arc of 340 degrees, 10 kΩ of electric resistance, and 2% as for linearity accuracy.

#### 2.2.2. Control Strategy

The control strategy is planned considering the main characteristics of the pneumatic muscle: compliance and non-linear behaviour. Furthermore, the system presents non-linearities due to the presence of two links which involve a dynamics depending on the current configuration of the system. In [97], an upper limb rehabilitation machine with pneumatic muscles is presented and it is demonstrated that a classic PID controller is not more suitable for linear systems. Then, a Fuzzy Logic Controller (FLC) was evaluated. The most notable feature of an FLC is the “translation” of fuzzy linguistic rules and measurements into non-linear mapping. An FLC can be adjusted through practical observation or experience, almost ignoring the complexity of the installation. An FLC can face complex systems with relative ease, still providing robustness and logical interpretability because it can deal with uncertainty (system’s variations, sensors noise) being defined in an uncertain manner. For this reason, fuzzy logic was chosen to implement a control system. 

**Figure 8:**
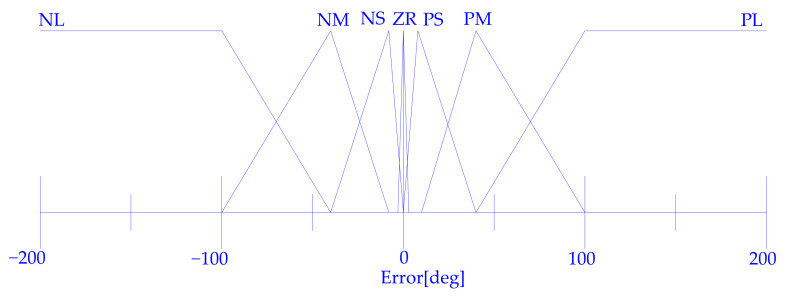
The membership functions for the fuzzyfication of the shoulder and elbow angular error.

Therefore, as a first step, a closed loop position and trajectory control system based on fuzzy logic is implemented. To define this system, it is not important to know if the relationship among internal pressure, contraction, and traction force is linear or not, but only the qualitative connection. It allows for the description of the qualitative behaviour of the controller by mean of linguistic rules whose quantitative meaning is defined by the membership functions shape, using in-house-developed control software with a fuzzy routine in C. The PWM driving allows the conductance of the valves to be continuously ruled between zero and fully open valve conductance. Hence, the control can compute the duty cycle for the valves. For one couple of muscles, the control system computes a parameter in the range [−1, +1], used to drive the 2 valves. When the specified parameter value is negative, the exhaust valve is driven with a duty-cycle equal to the absolute value of the specified parameter. Positive values drive the supply valve with a duty cycle equal to the specified parameter. For the other couple of muscles, in the antagonist position, what is said before is applied on the contrary: if, for a couple of muscles the exhaust valve is driven, for the other one, the supply valve is driven and vice versa. In Figure 8, the fuzzyfication of the shoulder and elbow joint angular error is reported. 

**Figure 9:**
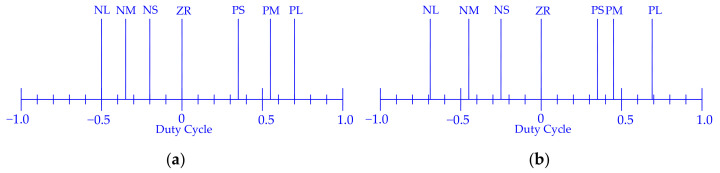
Defuzzyfication for elbow joint for flexion muscles (**a**) and extension muscles (**b**).

In Table 3, there are the fuzzy rules and in Figure 9, as an example, the defuzzyfication graphs for the elbow are presented.

With the described modalities, it is also possible to control the single joints simultaneously. For the command of trajectories within the working volume, it is necessary to define a trajectory and derive the motion laws in the joint space.

#### 2.2.3. Inverse Kinematic Model

In order to be able to carry out experimental tests with trajectory tracking, the inverse kinematic model of the developed device was considered. Considering the transformation matrix from the local reference of link 2 to the base and expressing the position of the end of link 2 with respect to the base we have (Figure 1):

(4) T20=cosψsinψ0xF−sinψcosψ0yF00100001

By comparing Equation (4) with Equation (1) we obtain:

(5) $$\left\{\begin{array}{c} x_{F}{\;=\;L}_{2}{\cos\theta }_{1}{\cos\theta }_{2}{-\;L}_{2}{\sin\theta }_{1}{\sin\theta }_{2}{\;+\;L}_{1}{\cos\theta }_{1}\\ y_{F}{\;=\;L}_{2}{\cos\theta }_{1}{\sin\theta }_{2}{\;+\;L}_{2}{\sin\theta }_{1}{\cos\theta }_{2}\;{+\;L}_{1}{\sin\theta }_{1}\\ {\cos\psi \;=\cos\theta }_{1}{\cos\theta }_{2}{-\;\sin\theta }_{1}{\sin\theta }_{2}\\ {\sin\;\psi \;=\cos\theta }_{1}{\sin\theta }_{2}{\;+\;\sin\theta }_{1}{\cos\theta }_{2} \end{array}\right.$$

From system (5), considering the additional trogonometric formulas, it is possible to obtain:

(6) $$\left\{\begin{array}{c} x_{F}{=L}_{1}{\cos\theta }_{1}{+L}_{2}{\cos(\theta }_{1}{+\theta }_{2})\\ y_{F}{=L}_{1}{\;\sin\;\theta }_{1}{+L}_{2}{\cos(\theta }_{1}{+\theta }_{2})\\ {\psi =\theta }_{1}{+\theta }_{2} \end{array}\right.$$

The first two equations of system (6) can be transformed by mathematical developments based on a geometric approach to obtain the following expressions of θ_1_ and θ_2,_ made explicit as functions of x_F_ e y_F_:

(7) $${\cos\theta }_{2}=\frac{x_{F}{}^{2\;}{+y}_{F}{}^{2}{\;-\;L}_{1}{}^{2}{\;-\;L}_{2}{}^{2}}{{2L}_{1}L_{2}}\;\;\;\;\;\;\;$$

(8) $$\;{\sin\theta }_{1}=\sqrt{\frac{y_{F}{}^{2}L_{1}{}^{2}{+y}_{F}{}^{2}L_{2}{}^{2}\cos^{2}\theta_{2}{+x}_{F}{}^{2}L_{2}{}^{2}\sin^{2}\theta_{2}{+2L}_{1}L_{2}y_{F}{}^{2}{\cos\theta }_{2}{-\;2L}_{1}L_{2}y_{F}x_{F}{\sin\theta }_{2}{\;-\;2L}_{2}{}^{2}y_{F}x_{F}{\cos\theta }_{2}{\sin\theta }_{2}}{{(x}_{F}{}^{2}{+y}_{F}{}^{2}{)(L}_{1}{}^{2\;}{+L}_{2}{}^{2}{+2L}_{1}L_{2}{\cos\theta }_{2})}\;}$$

Using these expressions, it is possible to obtain the motion laws of the joints for any trajectory given as a sequence of points P(x_F_, y_F_).

## 3. Results

**Figure 10:**
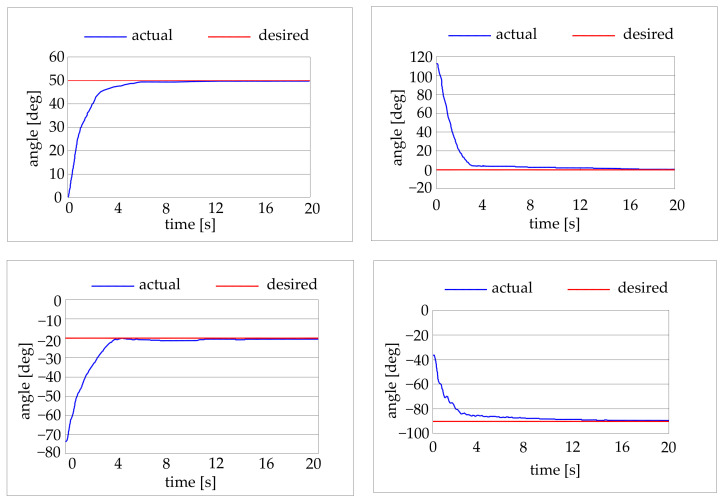
Results for step positioning of elbow joint (top) and shoulder joint (bottom).

**Figure 11:**
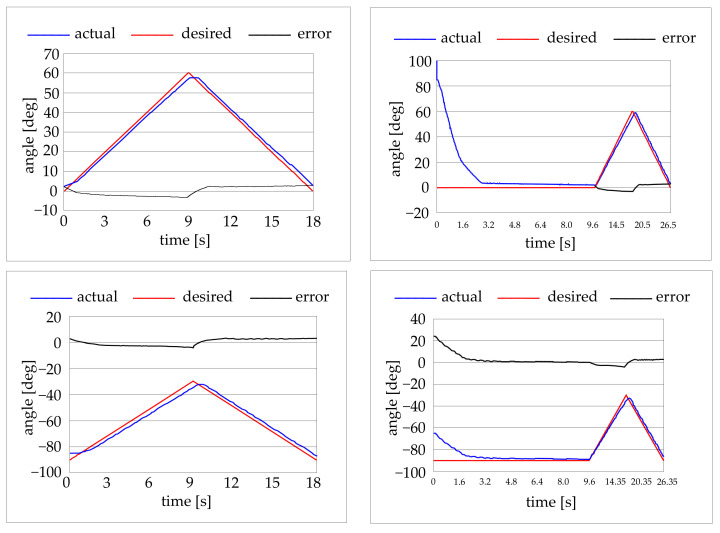
Results for trajectory tracking of elbow joint (top) and shoulder joint (bottom).

Three types of preliminary experimental tests were conducted. Some tests were carried out giving a step input at the control system and recording the robot behaviour as values of angular position vs. time. The target positions were chosen to obtain only the movement of one joint at a time. Some tests on the position accuracy of the elbow and of the shoulder were conducted for different angular positions of the joints. Then some target trajectories in the joint space were selected for the movement of one joint at the time. Finally, some target trajectories in the working volume were selected for the movement of both the joints at the same time. Figure 10 shows result of the former tests. Figure 11 shows results of the trajectory tracking tests in the joint space (one joint at the time). The trajectory starts from the rest position and far from the rest position.

**Figure 12:**
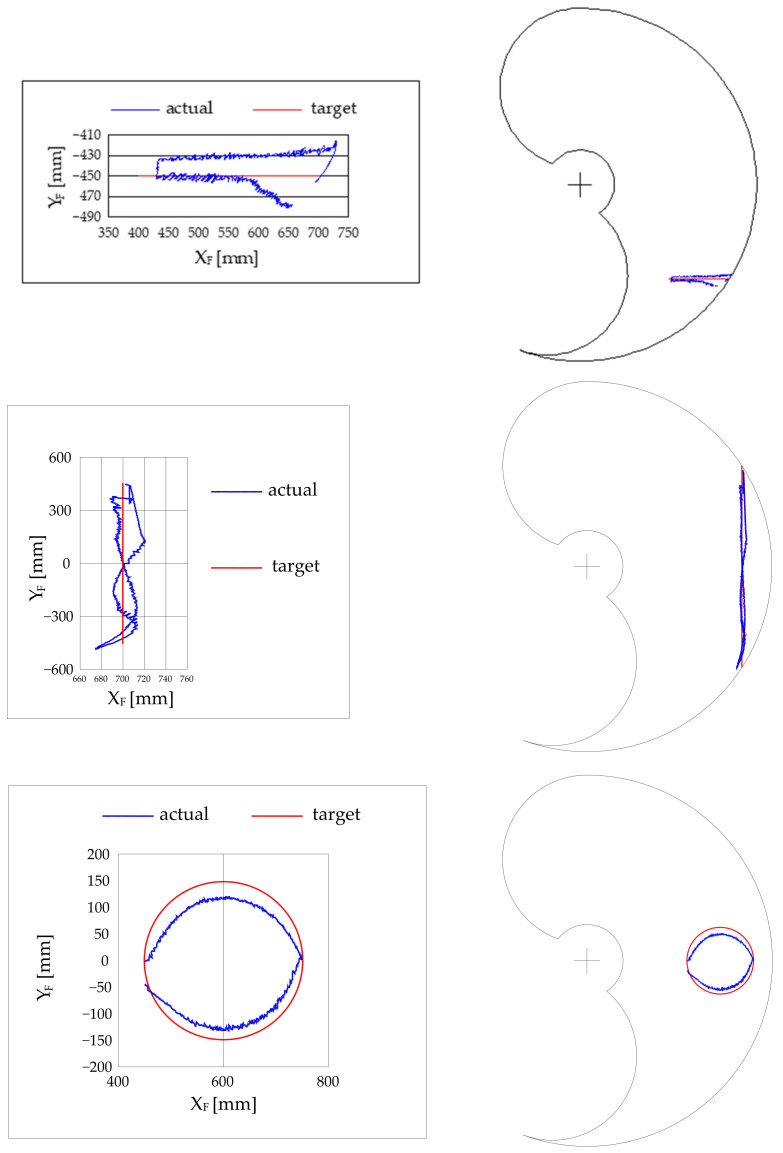
Results for trajectory tracking of a horizontal line trajectory (top), vertical line trajectory (middle), circular trajectory (bottom). The authors contend that although the kinematic domain was not totally explored, the carried-out exploration is sufficient to proof the concept.

The capability of the robot to follow a desired trajectory was also preliminary tested. Three trajectories were tested: a linear horizontal trajectory, a linear vertical trajectory, and a circular trajectory with a 300 mm diameter. Some tests results are in Figure 12.

## 4. Discussion and Conclusions

From the result shown in Figure 9, Figure 10 and Figure 11, it can be seen that as for the step tests the robot need from 4 to 6 s to reach the target position. It is interesting to remark that for application in the rehabilitation sector, the precision and the position accuracy of the robot are not critical parameters, and a low velocity is requested. The maximum absolute error is 1 degree for the elbow and 2 degrees for the shoulder.

As for the trajectory tests in the joint domain, with movements of one joint at the time, it can be seen that the absolute error is confined within the ±3 degrees range for both the joints.

As for the trajectory tests involving both the joints at the same time, it can be seen the absolute error is within the ±30 mm range.

**Figure 13:**
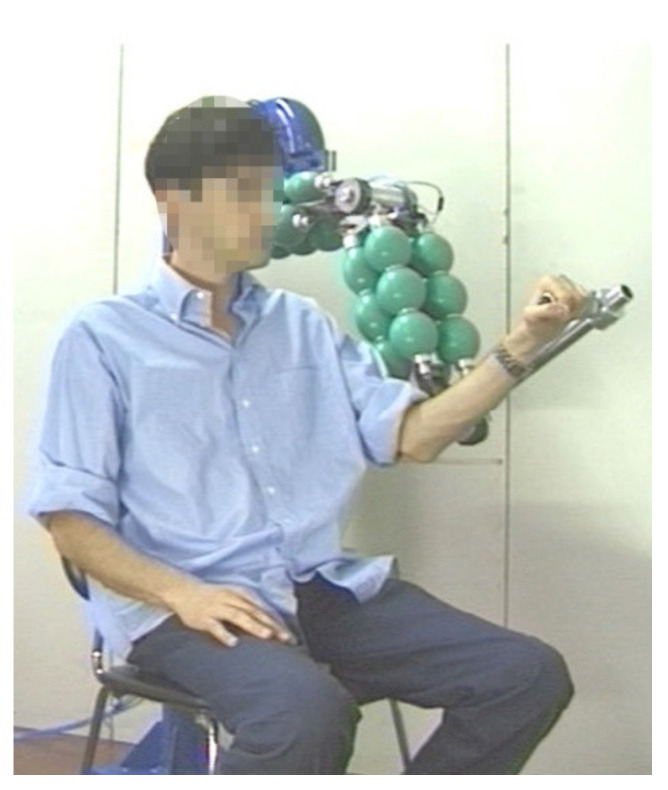
The machine is able to support and drive the limb of the user. The behaviour is smooth thanks to the compliance due to the pneumatic muscle actuators.

The 3 degree error on the shoulder joint results in an error of about 30 mm on the robot end. Although this error may be considered excessive for industrial applications, it is not excessive for a rehabilitation application. In fact, if we consider the value of 30 mm compared to the vertical dimension of the working volume equal to 1600 mm, this corresponds to a percentage error of 1.9%. From the point of view of the actuators, it should be noted that an error of 3 degrees, with a diameter of the transmission pulleys equal to 63.66 mm, corresponds to an error on the length of the pneumatic muscle equal to ±1.5 mm, which is a good value for a pneumatic actuator. On the other hand, observing the real trajectories in the working volume, we can see how these are certainly within the precision that a healthcare professional can guarantee by imposing movements on the user with his own limbs. Furthermore, the system is actually characterized by compliance as expected. The system can easily support the user’s limb and impose the reference trajectory. The imposed movement is smooth thanks to the softness of the machine. In Figure 13, a photograph taken during a test is shown.

Regarding the other performances, Table 4 shows the values assumed by the specification parameters. In the table, there is also a comparison with another design hypothesis that uses a conventional approach for what concerns the actuators, which are one of the characterizing aspects of this project. This is in order to better highlight any advantages brought about by the use of the technology proposed here concerning the actuators.

Comparison would be desirable with existing machines and on the basis of a common denominator, but, as mentioned in the introduction, there are no standards. Therefore, the types, albeit strictly in the field of upper rehabilitation machines, are many. In fact, there are more than 120 upper limb rehabilitation devices of which only 19 treat shoulder and elbow rehabilitation [94]. Of these, none have the same kinematic architecture and only two come close to the developed robot. Of these two, one has unconventional actuators specially designed and manufactured for the purpose, another has conventional actuators, ie electric motors, but the powers involved do not seem comparable and therefore the comparison would not be on a congruent basis; in any case, no quantitative information is available for this purpose.

For these reasons, the comparison was made considering a hypothesis with the same kinematic architecture, the same dimensions and kinematic domain and the same construction solutions adopted for the current project, but with electric motors.

The hypothesis is based on the use of brushless electric motors with inexpensive gearboxes with worm and helical wheel. In addition, 0.9 Nm motors are considered that require gearboxes with a transmission ratio of 10 for the elbow joint and 35 for the shoulder joint, respectively. These reducers are non backdrivable and, for this reason, an intervention is necessary to introduce compliance. This can be obtained by applying a suitable flexible mechanical device [98] between the actuator and the joint, or by controlling the interaction (by means of impedance control of the arm). In this second case, force sensors interposed between the motor and the joint being moved are required and in any case the solution may have reliability problems due to the inevitable delays of the control system with respect to the mechanical system. Both solutions were considered to involve a cost of EUR1000 for each joint. Also required are drives, a power supply, power cables, control cables, and a CAN interface.

By the results shown, it is possible to state that the feasibility of the project here proposed is demonstrated, resulting in an outperforming of a conventional solution. Future developments will concern the implementation of other types of functions with active patient possibly through the use of other control techniques such as the Generalized Predictive Control particularly suitable for non-linear systems. Furthermore, a database-based system will be implemented for monitoring the patient’s evolution according to rehabilitation programs. Clinical trials for the complete validation of the project will follow.

## Figures and Tables

**Table 1:** Requested torques at the joints of the robot.

	Maximum Torque [Nm]	Minimum Torque [Nm]
Shoulder joint	32.37	−22.16
Elbow joint	9.02	9.02

**Table 2:** Functional characteristics of the two versions of pneumatic muscle used in the robot.

	Length	Rest Diameter	Maximum Diameter	Maximum Force	Maximum Contraction	Number of Segments
Shoulder joint	400 mm	30 mm	90 mm	508 N	110 mm	4
Elbow joint	300 mm	30 mm	90 mm	142 N	90 mm	3

**Table 3:** Fuzzy rules table.

Position Error	NL	NM	NS	ZR	PS	PM	PL
Flexion muscles	NL	NM	NS	ZR	PS	PM	PL
Extension muscles	PL	PM	PS	ZR	NS	NM	NL

**Table 4:** Characteristics of the machine and comparison with a brushless electric motors solution. The compliance in a nominal working configuration with the arm and forearm aligned.

Actuators	Compliance [mm/N]	Weight [N]	Footprint [mm^2^]	Cost [€]	Acceptability
SF Pneumatic muscles	✓ (4.67)	✓ 363	✓ 300 × 800	✓ 5580	✓✓
Brushless Electric motors	✓	✗ 453	✓ 300 × 800	✗ 11,050	✓

## Data Availability

Not applicable.
